# Less haste, more speed: Does delayed blood culture transport time lead to adverse incubation times or yield?

**DOI:** 10.1016/j.jinf.2025.106520

**Published:** 2025-07

**Authors:** Gavin Deas, Fergus Hamilton, Philip Williams

**Affiliations:** a Bristol Royal Infirmary, Marlborough Street, Bristol BS2 8HW, United Kingdom; b MRC Integrative Epidemiology Unit, Bristol BS6 5ES, United Kingdom

**Keywords:** Microbiology, Blood culture, *Streptococcus pyogenes*

## Abstract

**Background:**

Blood culture remains a vital diagnostic tool in the acutely unwell patient. The UK Standards for Microbiology Investigations (SMI) stipulate pre-analytical requirements that are aimed at increasing yield and reducing turnaround time. The requirement to load blood cultures on machines within 4 h has been shown to reduce turnaround time but limited evidence exists as to whether it improves yield.

**Methods:**

We extracted blood culture results, including organism growth, time to detection, location and time of sample collection from 4 hospitals in Southwest England. We then used mixed effects, Bayesian linear and logistic regression models to examine the effect of predictor variables like time to laboratory (TTL) and sent time on the response variable of growth or time to detection. We fitted generalised additive models to explore non-linearity.

**Results:**

449,191 culture sets were analysed, 398,077 of which had enough data to include in the final analysis. 37,255 sets flagged positive (9.36%) of which 21,330 were considered pathogens. Our primary analysis identified a small decrease in yield with each hours delay in loading (0.997 (95%CrI 0.994–1.001)). This effect was largest in *Streptococcus pneumoniae, agalactiae* and *pyogenes*. In our analysis on time on the machine, culture sets spend 10.03 (95%CrI 12.66–7.31) minutes less on incubators for each hour delay. Neither anaerobes nor yeasts showed any loss of recovery from increasing TTL. There was no convincing non-linearity in either of these effects.

**Conclusion:**

There is a marginal loss of growth for every hour a blood culture is left unincubated, with the loss of recovery of *Streptococcus pyogenes* and other streptococci being most common. There was no evidence of a reduction in Gram-negatives, anaerobes, or yeasts. There was a small decrease in time to detection for delayed sets. This analysis suggests there may be marginal benefit in reducing time to load.

## Highlights

• The UK SMI stipulates that blood cultures should be loaded within 4 h of being taken.
• The effect on yield has not been determined for delayed loading.
• We found overall that there was a 0.3% loss of yield per hour of delay.
• There was a clinically significant loss of *Streptococcus pyogenes* at a 3.2% per hour delay.

## Background

Blood culture remains a standard and common method for diagnosing bacteraemic patients and is often used in the setting of the acutely unwell, undifferentiated patient groups.^1^ Although culture remains the gold standard, it is recognised that it is insensitive, with many patients remaining culture-negative despite clear clinical evidence of infection. Additionally, due to the requirement for culture and identification, results are often not available to clinicians for >24 h from admission. As such, many organisations have set standards for blood culture in an attempt to increase both the speed of turn around (time-to-positivity, TTP) and yield of blood culture.

In the UK, the Standards for Microbiology Investigation (SMI) for Sepsis and Systemic or Disseminated Infections includes recommendations to load samples onto an incubator within 4 h of collection,^2^ henceforth called time-to-load (TTL). This pre-analytical requirement for TTL goes alongside other requirements on the inoculum size, with volume requirements and bottle numbers to increase the clinical utility of the test. The evidence supporting the recommendation on TTL includes an effect on total time between collection and pathogen detection, termed the time to positivity (TTP).^3^, ^4^

Remarkably, the effect of reduced TTL on yield is still not clearly understood. It seems possible that earlier incubation of blood cultures could improve yield, particularly of pathogens that are temperature sensitive. However, some evidence supports pre-incubation as *decreasing* yield, a paradoxical result that has not been widely explored. Although studies^5^, ^6^ have been performed of in-vitro analysis of spiked blood cultures, it is not clear that these analyses reflect the reality of clinical microbiological sampling where microbiological load is often unknown, and patients may have received antimicrobials or other treatments that might alter microbial growth.^7^ As such, we performed a large (>300,000 blood culture bottles) analysis of the practical implications of altered TTL.

## Methods

### Setting and study inclusion

This is a retrospective observational analysis of the blood culture pathway in the Severn Pathology network. This network serves 4 hospitals in the Southwest of England, including 2 district general hospitals and 2 tertiary referral centres, they saw a combined 31398 patients in March 2024^8^ in their emergency departments and have an overnight bed base of 2664 beds.^8^ Each site has blood culture (BACTEC FX) incubation machines. All blood culture bottles sent to our laboratory from the 1st January 2017 to 30th June 2023 were analysed. Culture sets are taken in line with local antisepsis protocol and processed within the laboratory in line with national protocols outlined in UK SMI S12.

### Clinical data

**Fig. 1:**
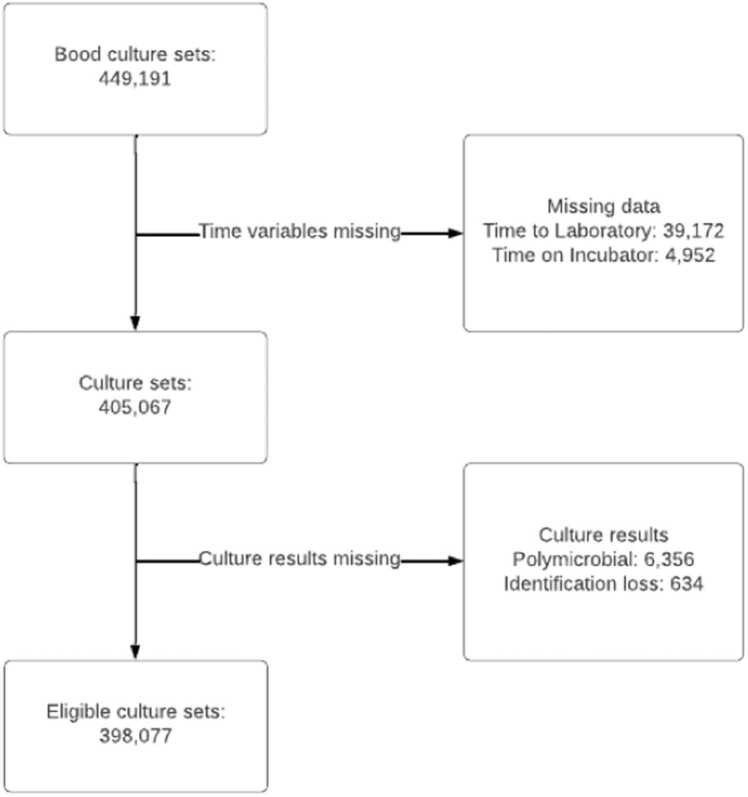
Flow chart of culture set inclusion.

We collected information on samples received on a) ward and hospital, b) timing of the blood culture (collection, loading, result, MALDI-TOF result), and c) date. The ICE (Integrated Clinical Environment™) requesting system was used to establish sample collection time. The BD BACTEC FX incubator collects loading time and time to detection (TTD) automatically. Duplicate sets of blood cultures were removed, and the quickest time to detection was treated as the time spent in the incubator. Organisms were classified into pathogenic organisms or contaminants by the authors, based on whether they pose clinical significance to patients on a population basis. Although any organism can be considered pathogenic or a contaminant given the right circumstances, the authors chose organisms that pose significant clinical disease and form the mainstay of clinical microbiological work (Fig. 1). This list is available in Supplementary Material 1.

### Statistical methods

Descriptive statistics of the sources, time to detection, loading, and organism are provided. Naïve linear and logistic regression analysis, unadjusted and adjusted Bayesian modelling were performed and generalised additive models were used to fit splines to explore the presence of non-linearity. One potential problem with analysing time to loading is that estimates of growth are biased by the physical location of wards and hospitals, and whether samples were taken during laboratory operating hours. A ward that we might expect to have a high positivity rate, for example, an intensive care unit, being far from the laboratory, would produce a paradoxical effect of an increased time to laboratory correlating with positive culture. To overcome this, we built a mixed effects model that accounted for ward and site, with wards nested within hospitals. Formally, we allowed a random intercept for each ward, nested within each hospital, essentially allowing each ward to have its own baseline positive rate. We then ran logistic regression (for yield) and linear regression (for time to detection), assuming a fixed effect for both (i.e. that the effect on yield of delay was the same across wards and sites). There are 8 sites: each of the 4 hospitals was divided into emergency departments and inpatient groups, and there were 107 wards in total. Due to the large number of wards and to allow model fitting, we used a Bayesian model with weakly informative priors. Statistical analysis was performed using R 4.2.2 utilising the CRAN package brms.^9^ The code is supplied in Supplementary Material 2.

The logistic/linear regressions models chosen took the form *(growth ∼ TTL* + *working day* + *year* + *(1|site/ward)* and (*TTD* ∼ *TTL* + *working day* + *year* + *organism* + *(1|site/ward)*, where *working day* was a binary variable of the sample being taken between 0900–1700. This was used on the dataset with all isolates and then subsequently on a subset with pathogenic isolates only. Pathogen-specific analysis for the most common isolates and for anaerobic and fungal growth was also eperformed.

### Ethics

All data used in this study was anonymised at source on extraction from the clinical systems. No patient identifiable information was ever extracted, simply the ward, location, time of sample, and whether it was positive (including the organism isolated). As such, ethical approval was not required.

## Results

### Descriptive statistics

**Fig. 2:**
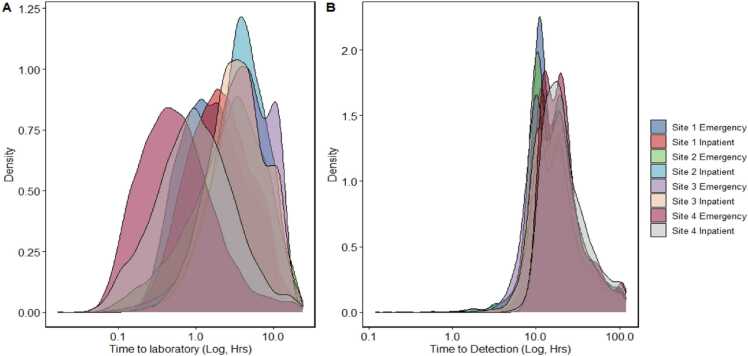
A) Density plot showing logarithmic distribution of Time to laboratory per site. B) A density plot showing time to detection per site.

449,191 sets were collected. This averaged out at 189.45 sets per day and 71.12 sets per 1000 bed days. Duplicates, entries with data loss for the loading time, time on machine, organism identification, or polymicrobial culture samples were removed, leaving 398,077 sets. 37,255 (9.36%) sets flagged positive, of which 21,330 were considered pathogenic and 15,925 contaminants, with 14,752 of the contaminants being coagulase-negative staphylococci. The mean Time to Laboratory (TTL) was 4.07 h (median 2.92 h; Q1 1.35 h; Q3 5.52 h). A density plot of total cultures received is shown in Fig. 2, broken down by site which clearly showed that the time to laboratory was different across differing sites.

Table 1 highlights a problem with naïve regression without accounting for ward structure. Emergency Departments have a very high level of positivity, as expected, but in one setting (Site 4 Emergency), the mean TTL is extremely low, as the laboratory is very closely co-located with this Emergency Department. A naïve regression analysis would identify this as suggesting a decreased TTL leads to increased yield, but this may just reflect the colocation of this laboratory and Emergency Department. In contrast, the Emergency Department in Site 3 has the highest TTL and a very high rate of positivity: a naïve analysis here would identify the opposite association.

**Table 1:** Top 25 sources for blood cultures, showing mean, median, standard deviation, 1st and 3rd quantile for the time to laboratory. The coefficient of variance, number of cultures received and the positivity rate for that site.

Source	Mean	Median	SD	Q1	Q3	CoV	N	Rate
Site 3 Emergency	5.10	3.88	3.89	2.12	7.28	0.76	32008	0.13
Site 1 Emergency	2.51	1.57	2.57	0.80	3.22	1.03	22784	0.15
Site 2 Emergency	4.12	2.85	4.04	1.22	5.63	0.98	22152	0.13
Site 4 Emergency	1.15	0.50	2.25	0.25	1.07	1.96	18185	0.13
Site 2 Haematology	5.66	4.45	3.90	2.82	7.72	0.69	14211	0.07
Site 1 ITU	5.59	4.63	3.99	2.68	7.55	0.71	13890	0.11
Site 2 Paediatric Emergency	3.87	2.87	3.40	1.45	5.22	0.88	13640	0.08
Site 1 Emergency	3.62	2.60	3.25	1.27	5.10	0.90	8871	0.09
Site 1 AAU	2.98	1.95	2.90	1.00	3.88	0.97	8752	0.08
Site 2 Haem/oncology	5.32	4.18	3.81	2.70	7.10	0.72	7458	0.07
Site 3 Haematology	4.45	3.28	3.57	1.97	5.85	0.80	7063	0.10
Site 3 ITU	6.05	4.83	4.25	2.82	8.38	0.70	6157	0.09
Site 3 MAU	5.11	3.80	4.05	2.10	7.15	0.79	5727	0.10
Site 2 CICU	4.24	3.38	3.44	1.68	5.77	0.81	5663	0.07
Site 2 Paediatric oncology	4.59	3.70	3.60	1.95	6.08	0.78	5609	0.07
Site 2 Delivery suite	4.90	4.08	3.44	2.35	6.58	0.70	5555	0.03
Site 1 AAU	3.06	1.95	3.12	0.95	4.07	1.02	4881	0.08
Site 3 Emergency	6.55	5.35	4.41	3.10	9.33	0.67	4400	0.13
Site 1 Delivery suit	3.83	2.77	3.23	1.48	5.32	0.84	4090	0.04
Site 1 Nephrology	4.32	2.85	4.09	1.50	5.72	0.95	3894	0.11
Site 2 NICU	4.98	4.14	3.45	2.45	6.60	0.69	3732	0.08
Site 2 Medical ward	4.43	3.57	3.51	2.02	5.65	0.79	3668	0.08
Site 3 SAU	4.06	2.82	3.61	1.55	5.31	0.89	3546	0.08
Site 3 Paediatrics	4.77	3.45	3.83	1.80	7.01	0.80	3411	0.07

### Decreased TTL leads to a marginal increase in yield in both unadjusted regression and in mixed effects models

The logistic regression model of (*growth ∼ TTL)* produced an OR for growth from time to laboratory of 0.987 for pathogenic isolates. For the pathogenic isolates the unadjusted, Bayesian, regression model (*growth ∼ TTL)* produced an Odds Ratio of 0.986 (95% credible interval 0.983–0.990). This can be interpreted as about a 1.4% decrease in yield for each hour delay.

In contrast, our mixed effects Bayesian regression model had an OR of growth of 0.997 (95%CrI 0.994–1.001) per hour: e.g. a decrease of around 0.3% per hour for each delay. This likely reflects the issues discussed earlier: the physical location of wards leading to biased estimates.

**Fig. 3:**
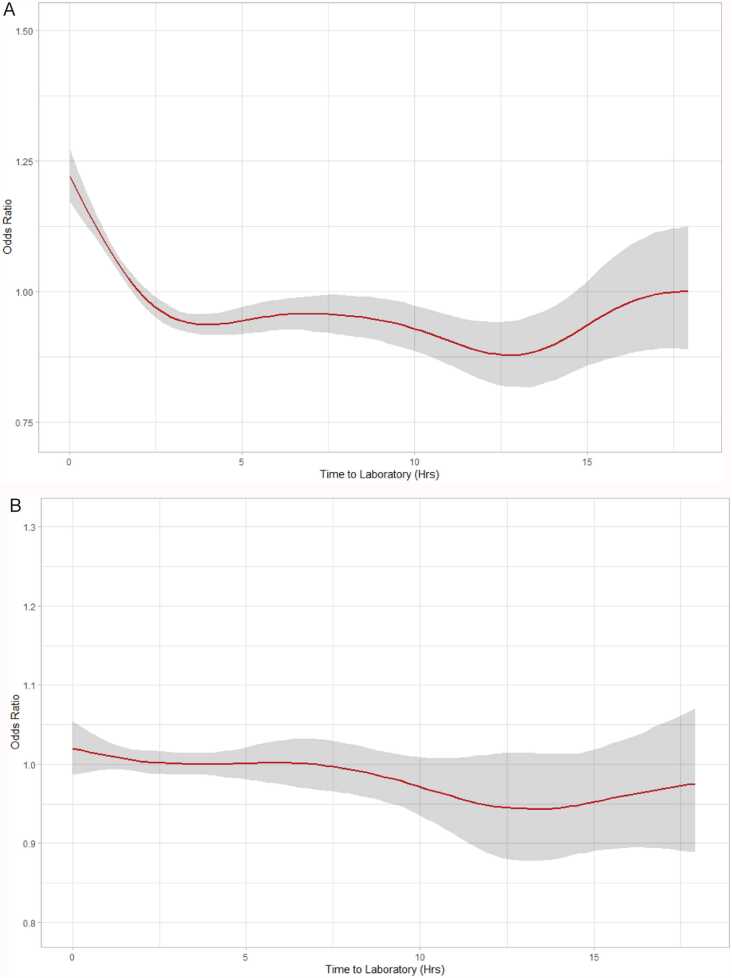
A) Generalized additive model plot of the estimate against time to laboratory for growth with 95% credible intervals. B) Generalized additive model plot of estimate against time to laboratory for growth with 95% credible intervals, adjusted for location, year, and working day.

**Fig. 4:**
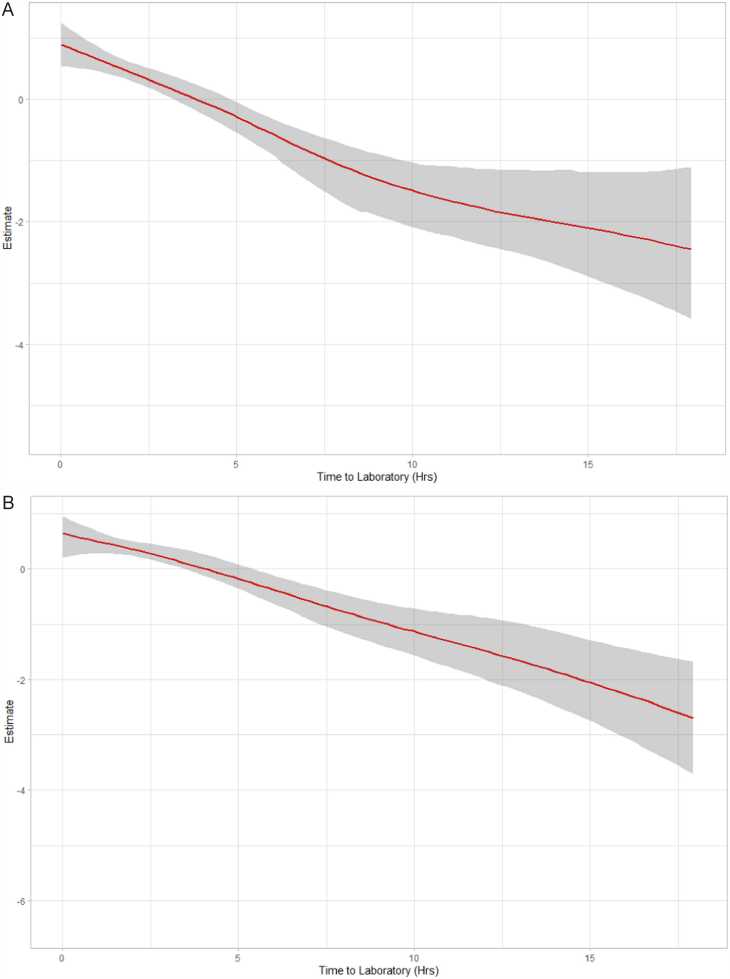
A) Generalized additive model of time to laboratory on time to detection with 95% credible intervals, without adjustment. B) Generalized additive model of time to laboratory on time to detection with 95% credible intervals adjusted for location, working day, organism and year.

We then performed non-linear modelling to show this and identify if there was a threshold effect. The unadjusted model is shown in Fig. 3A, and mixed-effect adjusted model in Fig. 3B. In the unadjusted model, there was clear evidence of non-linearity and even non-monotonicity with a large decrease in yield over the period 0–4hrs, but then almost no effect until ∼12 hrs, with a subsequent decrease in yield when the TTL increased after 12 h. In contrast, when accounting ward-level effects and time in the mixed-effects model, we see no evidence of non-linearity nor a threshold effect, and the data was consistent with a small, linear effect in reducing yield (Fig. 4).

### Loading blood culture bottles quickly leads to proportionately less time on the machine

The unadjusted Bayesian model *(TTD ∼ TTL)* identified that for every hour loaded earlier, there was an increase of 0.21 h (95%CrI 0.16–0.27) of time on the machine. In the mixed effects model, we saw a similar effect: 0.18 h (95%CrI −0.237 to −0.130). This suggests that for every hour increase in time to the laboratory, there is around 10 min less time spent on the incubator. As the total time to positivity is TTD + TTL, this means that some of the benefit of loading earlier is reduced by longer time on the machine, and is consistent with bacterial growth occurring prior to loading, whilst the culture set is in transit, but at a reduced rate. We did not identify any evidence of non-linearity, as expected.

### The effect on yield is dependent on the species of microorganisms: with a large effect on Streptococci including S.pyogenes

It is well established that different organisms have differing growth rates in culture, and it may be that the effect of delayed loading on yield is species-specific. Table 2 shows the list of isolates with greater than 250 occurrences with the mean time to laboratory and time on the machine, exclusive of contaminants. *E*. *coli* was the most common pathogen.

**Table 2:** Table of the isolates occurring more than 250 times, exclusive of contaminants, with mean TTL, median TTL, coefficient of variance and number of isolates.

Organism	Mean TTL	Mean TTD	SD TTL	CoV	N
*Escherichia coli*	3.34	13.93	2.84	0.85	6261
*Staphylococcus aureus*	3.34	19.93	2.70	0.81	3526
*Klebsiella pneumoniae*	3.55	15.29	2.85	0.80	1107
*Streptococcus pneumoniae*	2.85	11.75	2.52	0.88	760
*Pseudomonas aeruginosa*	3.44	21.07	2.82	0.82	678
*Enterococcus faecium*	3.61	16.50	2.79	0.77	655
*Enterococcus faecalis*	3.45	16.35	2.84	0.82	532
*Streptococcus agalactiae*	3.31	10.49	2.83	0.86	469
*Streptococcus dysgalactiae*	2.97	10.78	2.96	1.00	457
*Enterobacter cloacae complex*	3.79	14.62	2.79	0.74	440
*Proteus mirabilis*	3.37	17.66	2.87	0.85	423
*Streptococcus pyogenes*	2.88	11.24	2.72	0.94	352
*Coliform spp*	4.11	14.21	2.81	0.69	317
*Klebsiella oxytoca*	3.53	15.54	2.78	0.79	304
*Streptococcus mitis/oralis*	3.87	15.77	2.97	0.77	283
*Serratia marcescens*	3.38	17.72	2.69	0.80	267

As we know that at the organism is not known at the time of the blood culture being taken, and that the laboratory management of blood cultures is generally independent of the clinical management, we should expect the time to loading to be broadly similar for each species. However, Table 2 demonstrates subtle differences in mean TTL, *S*. *pneumoniae, dysgalactiae* and *pyogenes* have the quickest mean TTL.

As this difference is not due to earlier loading, this is likely due to decreased yield with longer TTL in some species. For example, if a species cannot survive post 8 hrs before being loaded, then we will only get positive results for this species when the TTL is <8 hrs, lowering the mean TTL. We therefore repeated our above analysis, focusing on each pathogen using the same Bayesian approach for these common pathogens.

**Fig. 5:**
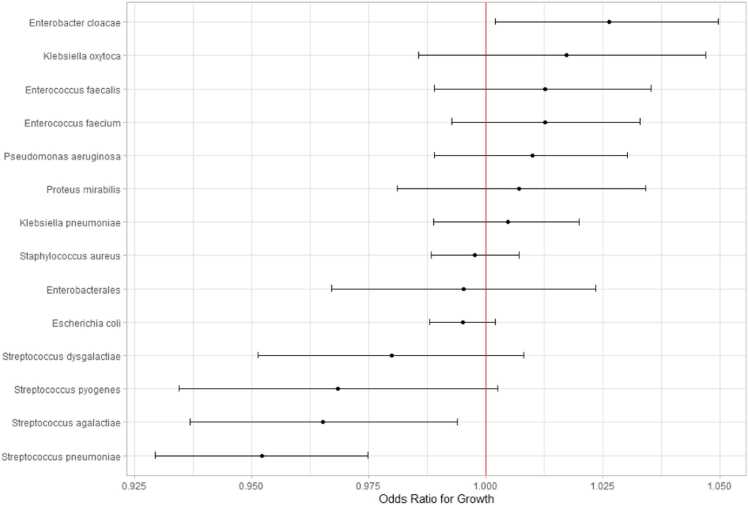
Forest plot for the estimate of growth per organism, adjusted for location, working day and year.

Fig. 5 shows a forest plot with 95% credible intervals for the most common isolates and their odds ratio of growth from the TTL. The organisms that showed the strongest effect for TTL reducing yield were *S*. *pneumoniae, agalactiae* and *pyogenes*.

The beta haemolytic streptococci appear to be significantly affected by delays in loading, with an odds ratio for *S*. *pyogenes* of 0.97 (95%CrI 0.93–1.00 per hour delay). This means that delaying loading *S. pyogenes* by 4 h might lead to a loss of around 20% of yield. *S*. *pneumoniae* may be explained by the known phenomena of autolysis.^10^

**Fig. 6:**
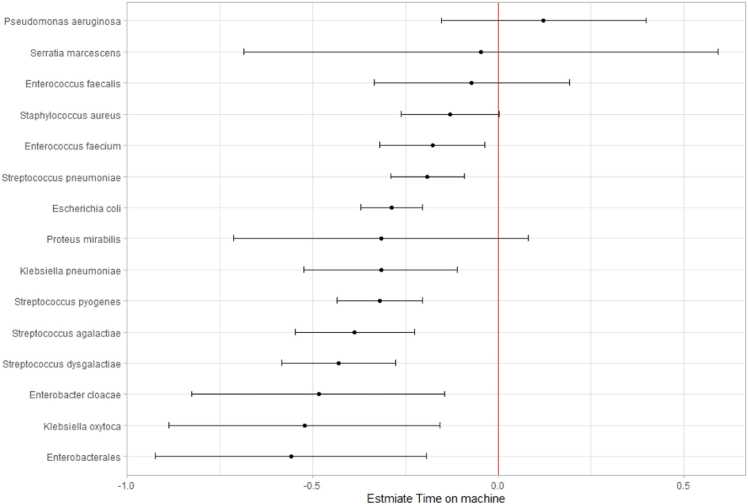
Forest plot of the estimate on isolates' time to detection from TTL.

Fig. 6 shows the relationship between the estimates on time to detection and mean TTL for each organism listed in Table 2. Unsurprisingly, most isolates fall to less than 0, meaning they spend less time on the machine for each hour they are delayed in getting to the laboratory*,* with the largest effects for the non-*E. coli* Enterobacterales and the beta-haemolytic Streptococci*.* However, for all species, estimates were larger than −1: the bacterial growth pre-loading was never fast enough to mean that delayed loading had no effect on the overall time to positivity.

Considering specific groups of pathogens, there were 518 fungal isolates with a mean TTL of 4.06 h and a median of 2.96 h. The adjusted model for yeasts produced an OR for growth from TTL was 1.01 (95%CrI 0.98–1.03). The estimate of TTL on time to detection was 0.003 (95%CrI - 0.003–0.01). Both indicating that yeast growth or time to detection was unaffected by TTL. Finally, considering the effect of time to laboratory on anaerobic bacterial growth, there were 629 anaerobic isolates, with a mean TTL of 3.67 h and median TTL 2.62 h. The OR for growth from TTL was 0.98 (95%CrI 0.95 – 1.00), and similarly, the OR for growth within the working day 0.98 (95%CrI 0.84–1.16).

## Discussion

This model of blood culture loading times showed that a longer time to laboratory (TTL) was associated with a lower rate of positivity, with an OR of 0.997 (95%CrI 0.994–1.001) for each hour, at the UK SMI standard of 4 h, the OR for growth is 0.987. This was consistent by considering all and pathogenic isolates. The loss of recovery of S*treptococcus pyogenes, pneumoniae* and *agalactiae* was most affected by the TTL variable. The unique feature of *S. pneumoniae* exhibiting autolysis may explain its estimate and loss of growth within delayed blood culture bottles. The loss of *S. pyogenes* is of both clinical and public health concern, a loss of up to 20% if cultures arrive in the laboratory less than 4 h from sampling. Invasive *S. pyogenes* is a severe infection and can lead to seeding to distant sites, requiring prolonged antibiotics and multiple surgical interventions. Most empirical guideline would seek to treat invasive *S. pyogenes* in a septic patient with gram positive antibiotics, and it remains reliably susceptible to penicillin.^11^ In patients who are difficult to obtain cultures from, such as paediatric or the immunocompromised, these results may be of increased importance, to prevent the pursuit of alternative causes for their clinical presentation; and, for the sake of stewardship, knowing the organism would allow for narrow-spectrum antimicrobials. The loss of invasive *S. pyogenes* isolates is of grave concern for the wider community, as missing the rates of circulating pathogenic strains could contribute to hidden pandemics. Regarding the UK SMI pre-analytical requirements, this data overall provides weak motivation for clinical teams to expedite their sample delivery. Whether this effect is worth investing in infrastructure to remove is debatable; it is well known that other factors have effects on yield that are far greater than that described here, principally blood volume.

Venturelli et al. published an observational study on time to laboratory and positivity rates in 2017. They reported the results of 50,000 culture results and found that they had an OR of 0.997 for growth for each hour in delay and an OR of 0.84 for growth for cultures taken outside of working hours; pathogens were equally affected across their study. Their laboratory only accepted culture sets in working hours but otherwise worked in alike our own laboratory.^12^

The effect of TTL on time to positivity and time to detection suggests that cultures spent less time on the incubators if they were loaded with a longer delay with a negative coefficient of –0.183, around 10.8 min quicker per hour delay. This is different from previous publications and the sources cited for the UK SMI, where a coefficient of greater than 0 is described, suggesting that for every hour delay, there is an increased TTD. This could be explained by the relatively short delay times here compared to the other studies, which looked at up to 48 h of delay and difference of 12 h or more between groups; or that the bottles here are subject to low level incubation whilst on the wards in our group that was not the case in the published groups, a factor that cannot be accounted for.^13^

The strength of this model is that it accounts for different patient populations within the hospital by nesting wards within hospital sites in the multilevel Bayesian model, in this way we also account for the effect of baseline positivity areas being different distances to the laboratory, adjustment in this manner means that OR for growth dramatically falls to very little compared to the unadjusted analysis. One unaccounted for factor is that blood cultures are an imperfect test, and it is assumed in clinical practice that even negative blood cultures in an antibiotic-naive patient may harbour pathogens. This means that we have no objective measurement for the presence of growth when looking at the various predictor variables, and as such, the forest plots showing the estimate of growth from time to laboratory for the most common pathogens are relative to each other and not to an objective reference organism.

## Conclusion

This analysis found that time to laboratory was independently and negatively correlated with a loss of yield at around 0.3% per hour, with a significant loss in some Streptococcal species. The Streptococcal species loss will require further investigation to understand the impact. This weakly supports the national guidance on blood culture pathways and the pre-analytical recommendation, and is the first publication to do so in this way, but there is no evidence that a cutoff of 4 h can be supported.

## Funding

PW is funded by the 10.13039/501100000265Medical Research Council grant MR/T005408/1. FH is funded by the 10.13039/100006662NIHR Clinical Lectureship programme.

## Declaration of Competing Interest

The authors declare that they have no known competing financial interests or personal relationships that could have appeared to influence the work reported in this paper.

## Footnotes

Appendix A Supplementary data associated with this article can be found in the online version at doi:10.1016/j.jinf.2025.106520.
