# Supplementary material for: Less haste, more speed: Does delayed blood culture transport time lead to adverse incubation times or yield?
Source: J Infect. 2025 Jul;91(1):106520. doi: 10.1016/j.jinf.2025.106520 (PMC12170351; doi:10.1016/j.jinf.2025.106520)
Supplement: Supplementary file 1 — Supplementary material [file mmc1.pdf]

Supplementary material 1

List of organisms considered pathogenic:

Enterobacterales
Pseudomonadales
Enterococcus
Staphylococcus aureus
Streptococcus pneumoniae
Beta-hemolytic streptococcus
Streptococcus anginosus
Streptococcus gallolyticus
Haemophilus influenzae
Haemophilus parainfluenzae
Streptococcus constellatus
Stenotrophomonas maltophilia
Streptococcus intermedius
Neisseria meningitidis
Corynebacterium striatum
Aerococcus urinae
Pasteurella multocida
Finegoldia magna
